# Probabilistic Safe Zone Mapping for S1 Screw Placement Using 1000 Lumbosacral CT Scans: A Study Protocol for a Bilateral, Two-Rater, Multi-Offset Anatomical Modeling Study

**DOI:** 10.3390/jcm15041323

**Published:** 2026-02-07

**Authors:** Nikolai Ramadanov, Robert Hable, Simon Zabler, Linus Michael, Roland Becker

**Affiliations:** 1 Center of Orthopaedics and Traumatology, University Hospital Brandenburg an der Havel, Brandenburg Medical School, Hochstraße 29, 14770 Brandenburg an der Havel, Germany; linus.michael@uk-brandenburg.de (L.M.);; 2 Faculty of Health Science, Brandenburg Medical School, 14770 Brandenburg an der Havel, Germany; 3 Faculty of Applied Computer Science, Deggendorf Institute of Technology, 94469 Deggendorf, Germany

**Keywords:** sacrum, sacral vertebrae, iliosacral fixation, screw fixation, computed tomography, anatomy, cross-sectional, probability, surgical planning

## Abstract

**Background/Objectives:** Safe placement of sacral vertebra 1 (S1) screws is essential in lumbosacral instrumentation and iliosacral fixation. Existing anatomical safe zones are largely based on averaged geometry and do not provide quantitative probability estimates for permissible deviations from an ideal entry point. This study aims to develop a probabilistic, computed tomography–based (CT-based) safe zone model for S1 screw placement. **Methods:** This retrospective imaging-based anatomical modeling study will analyze 1000 anonymized lumbosacral CT scans. A reproducible reference entry point will be defined on the lateral S1 projection, and bilateral offset-based virtual screw trajectories will be evaluated. Two independent raters will classify each trajectory as intraosseous or extraosseous. Probabilistic safety maps will be generated by aggregating binary classifications across offsets and directions. Interobserver reliability will be assessed using Cohen’s kappa, and anatomical influences will be analyzed using multivariable regression models. **Results:** The study is expected to generate continuous probabilistic safety maps illustrating the likelihood of intraosseous S1 screw placement across predefined offset distances and directions from the reference entry point. These maps are anticipated to demonstrate a gradual transition from high to low safety probabilities rather than a binary safe–unsafe boundary, and to identify anatomical factors influencing screw containment. **Conclusions:** This protocol describes a CT-based probabilistic modeling approach to S1 screw placement that aims to provide a more nuanced and quantitative definition of anatomical safe zones. If successful, the proposed method may improve preoperative planning and intraoperative decision-making by moving beyond averaged geometric constraints toward probability-informed screw placement.

## 1. Introduction

Posterior pelvic ring injuries and sacroiliac screw fixation have been extensively studied, with numerous investigations addressing anatomy, imaging, and technical accuracy. Early foundational work [1,2] established the principles of pelvic ring stability, the need for precise reduction, and the reliance on accurate screw trajectory to avoid neurovascular complications. Subsequent clinical series and technical studies have refined intraoperative imaging methods, demonstrating that fluoroscopic inlet, outlet, and lateral views remain essential for guidance [3,4,5,6]. These works collectively highlight that malpositioning continues to occur despite standardized imaging, underscoring the complexity of sacral anatomy.

CT-based anatomical studies have provided detailed morphometric analyses of the sacrum [7,8] and quantified corridor size, sacral variability, and the influence of patient factors such as sex and dysmorphism. More recent investigations expanded this understanding to include transiliac-transsacral screw corridors, angular tolerances, and patient-specific constraints [9,10,11]. Importantly, these studies consistently show that safe corridors are narrow, highly variable, and often not reliably identifiable using fluoroscopy alone. Several analyses [9,10,12,13] confirm that cortical breach remains common—even with experienced surgeons—and that radiographic safe zones do not consistently match true intraosseous anatomy.

Together, these studies [1,2,3,4,5,6,7,8,9,10,11,12,13] demonstrate three key gaps in current knowledge: (1) safe zones have been described only as geometric boundaries, not as probabilities; (2) existing studies rely on small sample sizes or idealized CT measurements; (3) no prior work quantifies how far an entry point may deviate in millimeter increments before cortical violation occurs.

Prior work has assessed screw trajectory and fixation safety using computational modeling, biomechanical testing, anatomical imaging, and navigation-assisted techniques [14,15,16,17,18,19]. These studies demonstrate that screw safety is highly dependent on local anatomy, bone quality, and trajectory-specific constraints, yet most rely on averaged geometry, static corridors, or device-specific guidance rather than quantifying tolerance to millimetric deviation [14,16,17,18,19]. Consequently, existing “safe zones” remain binary and idealized, limiting their ability to capture interindividual variability and to inform probabilistic risk assessment of cortical breach in clinical practice.

Against this background, the recently introduced Ramadanov–Zabler Safe Zone [20] represents an important conceptual advance. This pilot study developed a CT-based computational method to identify regions of higher bone density on the lateral sacral projection, producing the first reproducible, image-based definition of a safe entry region for S1 screw placement. Although derived from a single high-resolution CT model, the approach demonstrated feasibility, translational potential, and clinical relevance. It also highlighted the limitations of density-threshold segmentation in osteoporotic bone and the need for broader validation across anatomical variations.

However, by design, the pilot study [20] does not quantify interindividual variability, bilateral differences, or directional tolerance. It provides the conceptual framework, but not the probabilistic evidence needed for operative decision-making. Therefore, a large-scale, bilateral, multi-rater CT analysis of 1000 patients is the logical and necessary next step. Such a dataset allows: (1) probabilistic modeling of intraosseous screw safety, (2) mm-precise tolerance mapping in eight directions, (3) robust statistical evaluation using >320,000 datapoints, (4) validation of the Ramadanov–Zabler Safe Zone across a full anatomical spectrum.

This study will close the remaining evidence gap by establishing the first quantitative, probability-based safe zone map for S1 screw placement, enabling improved preoperative planning and reducing cortical breach risk in clinical practice.

## 2. Materials and Methods

### 2.1. Study Design and Data Source

The study protocol was preregistered in the Open Science Framework prior to data analysis (https://osf.io/xazdk/, accessed on 5 February 2026). The prospective publication of this protocol aims to ensure methodological transparency, prevent post hoc modification of analytical thresholds, and enable independent replication of probabilistic safe zone modeling in other CT datasets. This study protocol describes the predefined methodological framework and analysis plan; no imaging data have been analyzed at the time of submission. This study is designed as a retrospective, CT-based anatomical probability-mapping investigation. A total of 1000 anonymised high-resolution CT scans of the lumbosacral junction from adult patients (≥18 years) will be analyzed. Only CT datasets without prior instrumentation, fractures, tumors, or gross deformities of the sacrum will be included to ensure undistorted anatomical assessment.

### 2.2. Definition of the Standardized S1 Entry Point

**Figure 1:**
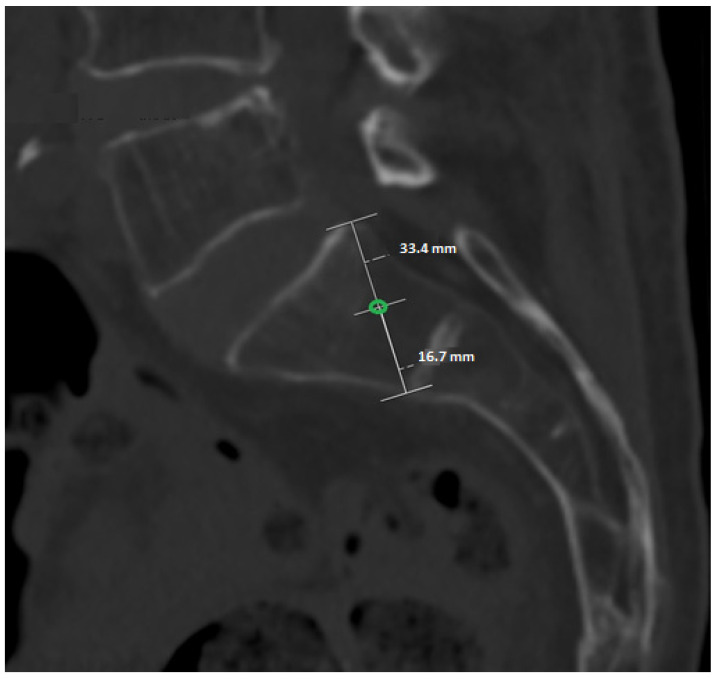
Lateral CT projection of the S1 vertebral body illustrating the standardized construction of the entry point. The dorsocranial and ventrocaudal cortical points are connected, and the midpoint of this line (green circle) represents the defined entry point (0 mm).

A reproducible S1 screw entry point will be defined on the lateral projection of the S1 vertebral body. On this view, the dorsocranial cortical point and the ventrocaudal cortical point of the S1 body will be identified and connected by a straight line. The midpoint of this line will serve as the standardized reference entry point (0 mm). This construction is applied identically in all CT datasets and for both sides (Figure 1). Figure 1 illustrates the standardized and reproducible anatomical construction used to define the reference S1 entry point, which serves as the fixed origin for all subsequent offset-based trajectory analyses.

### 2.3. Directional and Offset-Based Evaluation

From the standardized entry point, eight directional vectors will be assessed: dorsal, ventral, cranial, caudal, dorsal–cranial, dorsal–caudal, ventral–cranial, and ventral–caudal. Along each direction, ten offset positions will be evaluated in 1 mm increments from 1 to 10 mm, resulting in 80 distinct offset positions per side. All eight directional vectors are defined and evaluated within the standardized lateral S1 projection (2D), which reflects the clinically relevant fluoroscopic lateral view used intraoperatively.

### 2.4. Rating Procedure and Outcome Definition

Each CT scan will be evaluated bilaterally (left and right S1). Two independent raters will assess all offset positions. For every offset, the screw trajectory is classified dichotomously as fully intraosseous (InBone = 1) or cortical breach (InBone = 0). This results in 320 individual measurements per patient (2 sides × 2 raters × 80 offsets) and a total dataset of 320,000 observations across all CT scans. Although trajectory assessment is performed using a binary classification (fully intraosseous vs. cortical breach), the probabilistic nature of the model emerges from aggregating thousands of binary observations across offsets, directions, and individuals. This approach allows continuous safety gradients to be derived from discrete anatomical events, reflecting the true clinical endpoint of screw placement: cortical containment versus violation.

### 2.5. Data Structure

Patient-level data will be recorded in a dedicated dataset with one row per side, including CT identifier, side, age, sex, and basic S1 geometric parameters (height, width, and depth), as well as CT image quality indicators. Offset-level data will be stored in long format, with one row per measurement containing CT identifier, side, rater, direction, offset distance in millimeters, and binary intraosseous status. Detailed data extraction templates are provided in the Supplementary Materials (Supplementary Tables S1 and S2). The Supplementary Excel File represents a predefined data extraction template illustrating variable structure and coding; it does not contain patient data or sample observations.

### 2.6. Materials and Equipment

#### 2.6.1. CT Datasets and Imaging Parameters

This study will utilize anonymised high-resolution computed tomography (CT) datasets of the lumbosacral junction obtained during routine clinical imaging. Only adult patients (≥18 years) will be included. CT scans with prior lumbosacral instrumentation, fractures, tumors, congenital malformations, or severe degenerative deformities affecting S1 anatomy will be excluded. All included datasets must provide sufficient image quality to allow clear delineation of cortical bone boundaries at the S1 vertebral body.

Slice thickness, reconstruction kernel, and scanner manufacturer may vary across datasets, reflecting real-world clinical imaging conditions. This heterogeneity is intentionally accepted to enhance generalizability of the probabilistic safe zone model.

#### 2.6.2. Image Processing and Visualization Software

CT datasets will be analyzed using established medical image viewing and analysis software capable of multiplanar reformatting and precise distance measurements (e.g., OsiriX MD (Pixmeo SARL, Geneva, Switzerland; version 13.0 or later), Horos (Horos Project, Geneva, Switzerland; version 4.0 or later), 3D Slicer (Surgical Planning Laboratory, Brigham and Women’s Hospital, Boston, MA, USA; version 5.6 or later), or equivalent CE-certified DICOM viewers). All measurements will be performed in standardized orthogonal planes derived from the native CT data. No automated segmentation or artificial intelligence-based preprocessing will be applied.

#### 2.6.3. Hardware and Display Conditions

Image evaluation will be conducted on standard workstation computers equipped with calibrated medical-grade or high-resolution diagnostic displays. All measurements will be performed using digital measurement tools integrated within the imaging software.

#### 2.6.4. Data Handling and Documentation

Measurement results will be recorded in predefined electronic data extraction sheets developed specifically for this study. Patient identifiers will be removed prior to analysis. Offset-level data will be stored in long format, including CT identifier, side, rater, direction, offset distance, and binary intraosseous classification. Data management and statistical analyses will be conducted using standard statistical software R (R Foundation for Statistical Computing, Vienna, Austria; version 4.3 or later). 

### 2.7. Detailed Procedure

*Step 1: Dataset screening and eligibility*

All available lumbosacral CT datasets will be screened for eligibility. Datasets will be excluded if prior instrumentation, fractures, tumors, congenital anomalies, or severe degenerative deformities compromise the anatomical integrity of the S1 vertebral body. Image quality must permit reliable identification of cortical boundaries.

*Step 2: Standardized image orientation*

For each eligible dataset, multiplanar reformats will be generated. A standardized lateral projection of S1 will be established by aligning the anterior and posterior cortical margins of the S1 vertebral body to ensure consistent anatomical orientation across cases.

*Step 3: Definition of the reference entry point*

On the standardized lateral S1 projection, the dorsocranial and ventrocaudal cortical margins of the S1 vertebral body will be identified. The midpoint between these two landmarks will be defined as the reference entry point (0 mm offset).

*Step 4: Offset-based trajectory assessment*

From the reference entry point, parallel virtual screw trajectories will be evaluated at predefined offset distances in cranial and caudal directions. For each offset level, trajectories will be classified as intraosseous or extraosseous based on complete containment within cortical boundaries.

*Step 5: Binary classification and data recording*

Each assessed trajectory will be recorded as a binary outcome (intraosseous = 1, extraosseous = 0). For each dataset, side, offset distance, and direction, results will be documented in a predefined data extraction sheet.

*Step 6: Interobserver assessment*

Interobserver agreement will be formally quantified using Cohen’s kappa with 95% confidence intervals for the binary intraosseous classification. In cases of discordant ratings, a consensus decision will be reached through joint reassessment; if consensus cannot be achieved, a third senior assessor will adjudicate.

*Step 7: Aggregation and probabilistic modeling*

Binary trajectory data will be aggregated across all datasets. For each offset level, the proportion of intraosseous trajectories will be calculated to generate probabilistic safety profiles. These probabilities will form the basis for continuous safety gradient mapping across offset distances.

### 2.8. Statistical Analysis

The primary outcome is the probability of intraosseous screw placement for each predefined direction and offset, calculated as the proportion of CT scans in which the corresponding trajectory remains fully intraosseous. These probabilities will be used to generate continuous probabilistic safety maps.

Interobserver reliability will be assessed using appropriate agreement statistics. Bilateral differences between left and right sides will be analyzed using predefined comparative models. To evaluate the influence of anatomical variability, multivariable regression analyses incorporating S1 geometric parameters will be performed. All analyses will be conducted according to the predefined protocol using standard statistical software.

Continuous probabilistic safety maps will be generated by spatial interpolation of offset-specific probabilities and visualized as heat maps or contour plots overlaid on a schematic lateral S1 projection.

## 3. Expected Results

We expect that the probabilistic analysis will demonstrate a gradual decline in the proportion of fully intraosseous screw trajectories with increasing cranial and caudal offset distance from the predefined reference entry point. Rather than a binary safe versus unsafe boundary, the results are anticipated to reveal continuous safety gradients across offset levels, reflecting interindividual anatomical variability of the S1 vertebral body.

Specifically, offset ranges close to the reference entry point are expected to show high probabilities of intraosseous containment, while more extreme offsets will be associated with progressively lower probabilities and increased risk of cortical breach. We further anticipate asymmetric probability distributions between cranial and caudal directions, consistent with known anatomical differences in S1 morphology.

Interobserver agreement is expected to be high for binary trajectory classification, supporting the robustness and reproducibility of the proposed assessment framework. Sensitivity analyses are anticipated to confirm the stability of probability gradients across subgroups and measurement variability.

While the probabilistic maps are derived from anatomically normal, non-instrumented sacra, they are expected to be broadly applicable to standard trauma cases requiring S1 screw fixation. However, applicability to dysmorphic sacra or previously instrumented patients may be limited and will require separate validation in dedicated cohorts.

The probabilistic maps are expected to enable surgeons to identify S1 entry points with a quantified likelihood of fully intraosseous screw placement during preoperative CT planning. Intraoperatively, these probability gradients can be directly applied to the lateral fluoroscopic view to guide millimeter-level adjustments from the reference entry point based on predefined safety probabilities rather than binary safe zone boundaries.

Overall, the expected results aim to establish a reproducible, probabilistic safe zone concept that moves beyond rigid geometric margins and provides a more nuanced representation of screw placement safety. This framework is intended to support transparent methodological reporting and to facilitate subsequent validation and clinical application in future studies.

The primary results will be presented as probability heat maps and contour plots overlaid on a standardized lateral S1 schematic, allowing intuitive visualization of millimeter-level safety gradients for clinical interpretation.

## 4. Timeline and Review Status

The data extraction will commence immediately after acceptance of this study protocol in MDPI’s *Journal of Clinical Medicine*. The entire study will be conducted as efficiently as possible, with anticipated completion approximately 3–6 months after initiation.

## Data Availability

All relevant data extracted and analyzed during this study will be available from the corresponding author upon reasonable request.

## Supplementary Materials

The following supporting information can be downloaded at: https://www.mdpi.com/article/10.3390/jcm15041323/s1, Table S1: General data extraction sheet (structure only; no data included). Table S2: Data extraction sheet for the planned InBone measurement (structure only; no data included).
